# A Dual Frequency Carrier Phase Error Difference Checking Algorithm for the GNSS Compass

**DOI:** 10.3390/s16121988

**Published:** 2016-11-24

**Authors:** Shuo Liu, Lei Zhang, Jian Li

**Affiliations:** Key Laboratory of Electronic and Information Technology in Satellite Navigation (Beijing Institute of Technology), Ministry of Education, School of Information and Electronics, Beijing Institute of Technology, No. 5 Zhongguancun South Street, Haidian District, Beijing 100081, China; sure@bit.edu.cn (S.L.); lijian_551@bit.edu.cn (J.L.)

**Keywords:** GNSS compass, dual frequency, error difference checking

## Abstract

The performance of the Global Navigation Satellite System (GNSS) compass is related to the quality of carrier phase measurement. How to process the carrier phase error properly is important to improve the GNSS compass accuracy. In this work, we propose a dual frequency carrier phase error difference checking algorithm for the GNSS compass. The algorithm aims at eliminating large carrier phase error in dual frequency double differenced carrier phase measurement according to the error difference between two frequencies. The advantage of the proposed algorithm is that it does not need additional environment information and has a good performance on multiple large errors compared with previous research. The core of the proposed algorithm is removing the geographical distance from the dual frequency carrier phase measurement, then the carrier phase error is separated and detectable. We generate the Double Differenced Geometry-Free (DDGF) measurement according to the characteristic that the different frequency carrier phase measurements contain the same geometrical distance. Then, we propose the DDGF detection to detect the large carrier phase error difference between two frequencies. The theoretical performance of the proposed DDGF detection is analyzed. An open sky test, a manmade multipath test and an urban vehicle test were carried out to evaluate the performance of the proposed algorithm. The result shows that the proposed DDGF detection is able to detect large error in dual frequency carrier phase measurement by checking the error difference between two frequencies. After the DDGF detection, the accuracy of the baseline vector is improved in the GNSS compass.

## 1. Introduction

The Global Navigation Satellite System (GNSS) compass is an attitude indicator based on the theory of carrier phase relative positioning [1]. It is also known as the GNSS attitude determination or GNSS heading determination [2,3]. It is inexpensive and does not have the problem of divergence compared with the traditional Inertial Navigation System (INS) attitude determination. In the early days, the carrier phase integer ambiguity resolution was the bottleneck of the GNSS compass. The integer ambiguity needed a long time to be initialized [4]. Nowadays, as the integer ambiguity resolution algorithms have developed, initialization is no longer a problem, especially after the Least-squares AMBiguity Decorrelation Adjustment (LAMBDA), Constrained-LAMBDA and Multivariate Constrained-LAMBDA methods were proposed [5,6,7]. Now, the GNSS compass is widely used in automobiles, ships, airplanes and many other applications [5,8]. 

Another key technique of the GNSS compass besides integer ambiguity resolution is how to improve its accuracy. It is proven that the GNSS compass accuracy is related to the carrier phase measurement quality for a certain baseline length [9]. Most of the GNSS carrier phase error sources are highly correlated over space and time. The correlated error sources, such as satellite clock error, receiver clock error, ephemeris error, ionosphere delay and troposphere delay, can be almost eliminated by the differencing technique in the GNSS compass model as the baseline length is short [10]. Multipath and carrier phase noise are uncorrelated error sources. They cannot be eliminated and are even enlarged by the differencing technique. They become the main error sources in the GNSS compass. Therefore, processing the multipath and carrier phase noise are the keys to improving the accuracy of the GNSS compass further.

The influence of the multipath is usually more severe than carrier phase noise. Currently, multipath can be mitigated using three processing strategies [11]: (1) the receiver antenna technique [12,13]; (2) receiver signal filtering [14,15]; (3) observation data processing [16,17,18,19,20,21]. Among them, observation data processing algorithms are often used by the GNSS receiver user. The recent observation data processing research is mainly focused on multipath estimating and multipath detecting. In multipath estimating algorithms, the approximate multipath is estimated according to the empirical information. The performance of these algorithms is related to how precise the estimated multipath is. The satellite elevation is the most representative estimating algorithm [16,17,18]. However, satellite elevation is not very precise for estimating multipath, as the multipath of the same elevation satellites may be very different according to the real environment. It makes the accuracy improvement not very obvious [18,19]. As for multipath detecting, the 3D surface model and sidereal filter algorithms are the representative ones [20,21,22]. The exact multipath can be obtained. These algorithms are relatively accurate, but the prior environment information must be modeled first. This is not suitable for most of the real engineering situations, as the environment data are not always available especially in unknown dynamic environments.

There is another type of error detection method based on Detection, Identification and Adaptation (DIA) theory. The representative method, namely Receiver Autonomous Integrity Monitoring (RAIM), is often used in civil aviation Single Point Positioning (SPP) [23,24]. As for carrier phase relative positioning, a similar method is called Carrier phase-based RAIM (CRAIM) [25,26]. These methods are based on one-dimensional test-statistic [27]. It has a good performance on false alarm and single error identification. However, it is not very suitable for multiple error identification in real engineering situations. Hence, many of the recent carrier phase relative positioning research works do not utilize any algorithms to process carrier phase error [28,29,30], as well as dual frequency or multiple frequency research [31,32]. It somehow will influence the accuracy, especially when some large error occurs.

The GNSS satellite broadcasts signals in multiple frequencies. Some well-known carrier phase combinations can be generated by combining different carrier phase measurement, such as Wide Lane (WL), Narrow Lane (NL), Ionosphere Free (IF) and Geometry Free (GF) [33,34]. The geographical distance in the GF combination is zero. The most common usage of the GF combination is cycle slip detecting and integer ambiguity resolution. In this work, we find out that the correlated carrier phase error sources are also nearly zero in the double-differenced GF combination for the GNSS compass model. The double-differenced GF combination only contains multipath and carrier phase noise. Therefore, we can use the double-differenced GF combination to detect whether the carrier phase measurement has large multipath and carrier phase noise error. Compared with the previous work above [20,21,22,25,26], it does not need prior environment information, and its performance is not influenced by multiple large errors. Therefore, it is more suitable for real engineering.

In this paper, we propose a dual frequency carrier phase error difference checking algorithm for the GNSS compass. The algorithm is focused on detecting large carrier phase error, which cannot be eliminated by the differencing technique. By subtracting the geographical distances in the dual frequency DD carrier phase, we can obtain the Double-Differenced Geometry-Free (DDGF) measurement. As the correlated errors are nearly zero in the short baseline GNSS compass model, the DDGF measurement only contains multipath and carrier phase noise. We estimate the theoretical magnitude of the multipath and the carrier phase noise. Then, the theoretical threshold is formed. Whether the DDGF carrier phase has large multipath or carrier phase noise is detected according to the theoretical threshold. The carrier phase measurement that does not pass the DDGF detection will be eliminated by weighted estimation.

The rest of this paper is organized as follows: in Section 2, the principle of the proposed dual frequency carrier phase error difference checking algorithm is described. Section 3 presents the theoretical performance of the proposed algorithm. In Section 4, the experiment results, including the open sky test, manmade multipath test and urban vehicle test, are demonstrated and discussed. The conclusions are presented in Section 5.

## 2. Dual Frequency Carrier Phase Error Difference Checking Algorithm

### 2.1. Double-Differenced Carrier Phase Measurement Model

GNSS carrier phase relative positioning needs two receivers, respectively named “base” and “rover”. The carrier phase measurement observed by the two receivers at a certain epoch can be written as [35]:

(1) $$\phi_{b,k}^{i}=\lambda_{k}^{-1}\left(r_{b,k}^{i}-I_{b,k}^{i}+T_{b,k}^{i}\right)+f_{k}\left(\delta t_{b,k}-dt_{k}^{i}\right)+N_{b,k}^{i}+\varepsilon_{b,k}^{i}$$

(2) $$\phi_{r,k}^{i}=\lambda_{k}^{-1}\left(r_{r,k}^{i}-I_{r,k}^{i}+T_{r,k}^{i}\right)+f_{k}\left(\delta t_{r,k}-dt_{k}^{i}\right)+N_{r,k}^{i}+\varepsilon_{r,k}^{i}$$

where ϕ is the carrier phase measurement (unit: cycle), λ is the carrier wavelength (unit: m), r represents the true geographical distance between the satellite and the receiver (unit: m), I is the ionospheric delay (unit: m), T is the tropospheric delay (unit: m), f is the carrier frequency (unit: Hz), δt is the receiver clock error (unit: s), dt is the satellite clock error (unit: s), N is the integer ambiguity (unit: cycle) and ε is the residual errors mainly including carrier phase noise and multipath (unit: cycle). Subscripts b and r respectively represent the base and the rover receiver. Subscript k is the identifier for different frequencies, and k∈1,2 for dual frequency case. Superscript i represents the satellite #i.

The Single-Differenced (SD) measurement model can be obtained by subtracting the base receiver’s measurements from the rover receiver’s measurements, namely Equations (1) and (2).

(3) $$\phi_{rb,k}^{i}=\lambda_{k}^{-1}\left(r_{rb,k}^{i}-I_{rb,k}^{i}+T_{rb,k}^{i}\right)+f_{k}\delta t_{rb,k}+N_{rb,k}^{i}+\varepsilon_{rb,k}^{i}$$

where the subscript “rb” represents the difference between the rover and base receiver. It can be seen that $dt_{k}^{i}$ as a common error is eliminated by single differencing.

Then, choosing the j-th satellite with the highest elevation angle as the reference and the Double Differenced (DD) carrier phase between the reference and other satellites can be obtained as follows:

(4) $$\phi_{rb,k}^{ij}=\lambda_{k}^{-1}\left(r_{rb,k}^{ij}-I_{rb,k}^{ij}+T_{rb,k}^{ij}\right)+N_{rb,k}^{ij}+\varepsilon_{rb,k}^{ij}$$

where the superscript “ij” represents the carrier phase measurement of the i-th satellite minus that of the j-th (reference) satellite.

$\delta t_{rb,k}$ is the same for different satellite, so it can be eliminated by double differencing. $I_{rb,k}^{ij}$ and $T_{rb,k}^{ij}$ are related to the baseline length [36]. In GNSS compass model (the baseline length is several meters), $I_{rb,k}^{ij}$ and $T_{rb,k}^{ij}$ are nearly zero. Therefore, Equation (4) can be rewritten as:

(5) $$\phi_{rb,k}^{ij}=\lambda_{k}^{-1}r_{rb,k}^{ij}+N_{rb,k}^{ij}+\varepsilon_{rb,k}^{ij}$$

$N_{rb,k}^{ij}$ is the DD integer ambiguity. It is the unknown cycles in the DD carrier phase. It can be resolved by various algorithms, and it is relatively easy for the GNSS compass, as the baseline is short. In this work, we choose the popularly-used LAMBDA method [6,37] to resolve the integer ambiguity and assume that the integer ambiguity in the following DD carrier phase is resolved and removed. The DD carrier phase after the integer ambiguity resolution can be expressed as:

(6) $$\varphi_{rb,k}^{ij}=\phi_{rb,k}^{ij}-N_{rb,k}^{ij}=\lambda_{k}^{-1}r_{rb,k}^{ij}+\varepsilon_{rb,k}^{ij}$$

where φ is the carrier phase without integer ambiguity.

Once the integer ambiguity is resolved, the DD carrier phase becomes a highly precise measurement including geographical distance, carrier phase noise and multipath. Geographical distance is the useful information to calculate the baseline vector. Multipath and carrier phase noise are the useless components that will influence the accuracy of the baseline vector. Therefore, processing the carrier phase noise and multipath are the key to improving the positioning accuracy further.

### 2.2. Double Differenced Geometry Free Detection

From Equation (6), it is clear that after the integer ambiguity is resolved, the DD carrier phase measurement contains the geographical distance, multipath and carrier phase noise in the GNSS compass model. Dual frequency DD carrier phase measurement has exactly the same geographical distance. We define the double-differenced geometry free (DDGF) measurement by subtracting the geographical distances in the dual frequency DD carrier phase:

(7) $$\begin{array}{cc} \Phi_{DDGF}^{ij} & =\lambda_{1}\varphi_{rb,1}^{ij}-\lambda_{2}\varphi_{rb,2}^{ij}\\ & =r_{rb,1}^{ij}-r_{rb,2}^{ij}+\varepsilon_{DDGF}^{ij} \end{array}$$

where $\Phi_{DDGF}^{ij}$ and $\varepsilon_{DDGF}^{ij}$ are the DDGF measurement (unit: m) and DDGF residual error (unit: m). The geographical distances of dual frequency DD carrier phase measurement are the same, namely $r_{rb,1}^{ij}=r_{rb,2}^{ij}$. Equation (7) can be rewritten as:

(8) $$\Phi_{DDGF}^{ij}=\;\varepsilon_{DDGF}^{ij}$$

**Figure 1:**
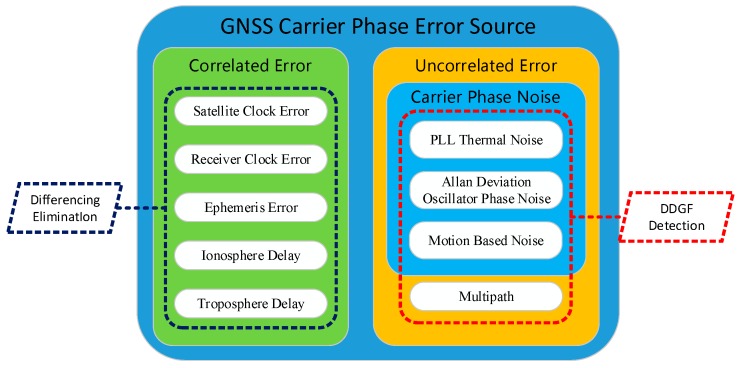
The relationship between the double-differenced geometry free (DDGF) detection and the GNSS carrier phase error sources.

From Equation (8), it is clear that DDGF measurement is supposed to be zero and only contains carrier phase error. The residual error is actually the error difference between two frequencies. Based on that characteristic, we design the DDGF detection by comparing the DDGF measurement and its theoretical estimation. Figure 1 shows the relationship between the DDGF detection and the GNSS carrier phase error sources. The GNSS carrier phase correlated error sources, such as satellite clock error, receiver clock error, ephemeris error, ionosphere delay and troposphere delay, can be eliminated by the differencing technique. DDGF detection is done to process the uncorrelated error sources, including multipath and carrier phase noise. In this work, we divide the carrier phase noise into three parts: Phase Lock Loop (PLL) thermal noise, Allan deviation oscillator phase noise and motion-based noise. Each part is estimated separately.

The theoretical magnitude of carrier phase and multipath can be estimated according to the receiver’s parameters. The thermal noise for an arctangent PLL is computed as follows [4,19]:

(9) $$\sigma_{PLLt}=\frac{1}{2\pi }\sqrt{\frac{B_{n}}{C/N_{0}}\left(1+\frac{1}{2TC/N_{0}}\right)}$$

where $\sigma_{PLLt}$ is the standard deviation of the PLL thermal noise (unit: cycle), $B_{n}$ is the carrier loop noise bandwidth (unit: Hz), $C/N_{0}$ is the carrier to noise power expressed as a ratio (unit: dB-Hz) and T is the pre-detection integration time (unit: seconds).

The Allan deviation oscillator phase noise of the third-order loop is [10]:

(10) $$\sigma_{A3}=\frac{160}{360}\frac{\sigma_{A}\left(\tau \right)f_{k}}{B_{n}}$$

where $\sigma_{A3}$ is the standard deviation of the Allan deviation oscillator phase noise (unit: cycle) and $\sigma_{A}^{2}\left(\tau \right)$ represents the Allan deviation.

The motion-based phase noise includes vibration-induced oscillator phase noise, dynamic stress error and reference oscillator acceleration stress error. The multipath and motion-based carrier phase noise is relatively hard to model. It depends on the motion of the receiver and the environment. In this work, we use the empirical value 2° [4]. According to the law of error propagation, the standard deviation of the carrier phase error can be expressed as:

(11) $$\sigma_{\varepsilon }=\sqrt{\sigma_{PLLt}^{2}+\sigma_{A3}^{2}+\sigma_{v}^{2}}$$

where $\sigma_{\varepsilon }$ represents the standard deviation of the carrier phase error (unit: cycle) and $\sigma_{v}^{2}$ is the variance of the multipath and motion-based carrier phase noise error (unit: cycle). Then, the standard deviation of the estimated DDGF measurement can be expressed as:

(12) $$\sigma_{\varepsilon ,DDGF}^{ij}=\sqrt{\sum_{k=1}^{2}\left({\left(\lambda_{k}\sigma_{\varepsilon ,r,k}^{i}\right)}^{2}+{\left(\lambda_{k}\sigma_{\varepsilon ,b,k}^{i}\right)}^{2}+{\left(\lambda_{k}\sigma_{\varepsilon ,r,k}^{j}\right)}^{2}+{\left(\lambda_{k}\sigma_{\varepsilon ,b,k}^{j}\right)}^{2}\right)}$$

where $\sigma_{\varepsilon ,DDGF}^{ij}$ is the standard deviation of the estimated DDGF measurement (unit: m). Usually, people believe the distribution should be within three-times of its standard deviation [10]. Therefore, the theoretical threshold of $\varepsilon_{DDGF}^{ij}$ without large multipath or carrier phase noise can be determined:

(13) $$T_{DDGF}^{ij}=3\sigma_{\varepsilon ,DDGF}^{ij}$$

If there is no large multipath or carrier phase noise, the magnitude of $\Phi_{DDGF}^{ij}$ should be within $T_{DDGF}^{ij}$; otherwise, there must be large residual error in the dual frequency carrier phase measurement. In that case, we eliminate the carrier phase of the corresponding satellite using weighted estimation.

For GNSS relative positioning, different carrier phase measurement can be given different weighting values according to the real environment, namely the weighted estimation [19]. The measurement is eliminated in case the corresponding weighting value is zero. Therefore, the corresponding weighting $p^{ij}$ is designed according to $\Phi_{DDGF}^{ij}$ and $T_{DDGF}^{ij}$:

(14) $$p^{ij}=\{\begin{array}{cc} 1 & \left|\Phi_{DDGF}^{ij}\right|<T_{DDGF}^{ij}\\ 0 & \left|\Phi_{DDGF}^{ij}\right|>T_{DDGF}^{ij} \end{array}$$

**Figure 2:**
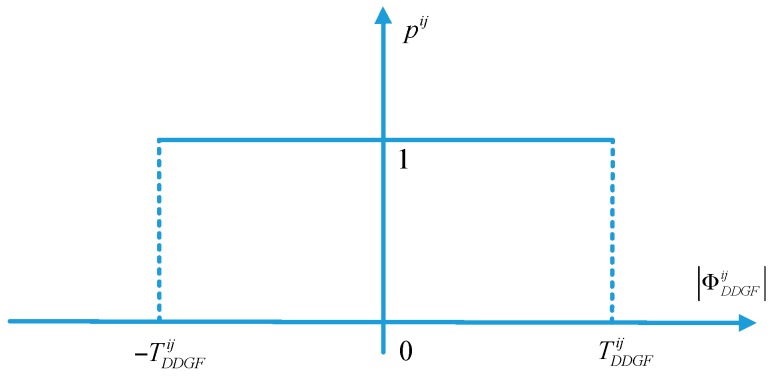
The illustration of the weighting value.

Figure 2 is the illustration of the weighting value according to $\Phi_{DDGF}^{ij}$ and $T_{DDGF}^{ij}$. 

From Figure 2, it is clear that the weighting is one when the DDGF residual error satisfies the theoretical threshold of the estimated carrier phase error, namely, $\left|\Phi_{DDGF}^{ij}\right|<T_{DDGF}^{ij}$. The weighting value is down to zero when the DDGF residual error is larger than the theoretical threshold.

### 2.3. Baseline Vector Estimation

The final product of GNSS carrier phase relative positioning is the baseline vector. The definition of the baseline vector is:

(15) $$b_{rb}=\left[\begin{array}{ccc} x_{rb} & y_{rb} & z_{rb} \end{array}\right]$$

where $b_{rb}$ is the baseline vector pointing from the base to the rover, $x_{rb}$, $y_{rb}$ and $z_{rb}$ are the coordinates of the baseline vector in Earth Centered Earth Fixed (ECEF) coordinate system (unit: m). 

The baseline vector is calculated by the DD carrier phase measurement. The relationship between the baseline vector and the DD carrier phase measurement is nonlinear. We expand the DD carrier phase using the Taylor series expansion method and reserve the first-order terms:

(16) $$\varphi_{rb,k}^{ij}=-\lambda_{k}^{-1}{\left(a_{r}^{i}-a_{r}^{j}\right)}^{T}b_{rb}$$

where $a_{r}^{i}$ is the normalized Line-of-Sight (LOS) vector pointing from the rover to the i-th satellite [21], and the LOS vector of the rover nearly equals that of the base under GNSS compass condition, i.e., $a_{r}^{i}\;=\;a_{b}^{i}$.

There are several DD carrier phase measurements of different satellites and different frequencies. We combine them as a vector. We exchange the place of the reference satellite #*j* to #1, then the satellite #1 is the reference satellite. Then, the DD carrier phase vector can be written as:

(17) $$\Phi_{rb}=\left[\begin{array}{cccccccc} \varphi_{rb,1}^{21} & \varphi_{rb,1}^{31} & \ldots & \varphi_{rb,1}^{q1} & \varphi_{rb,2}^{21} & \varphi_{rb,2}^{31} & \ldots & \varphi_{rb,2}^{q1} \end{array}\right]$$

where $\Phi_{rb}$ is the DD carrier phase vector and *q* is the total number of the available satellites.

In a similar way, the LOS of different satellites and different frequencies can be expressed as:

(18) $$H_{r}=-\left[\begin{array}{c} \lambda_{1}^{-1}{\left(a_{r}^{2}-a_{r}^{1}\right)}^{T}\\ \lambda_{1}^{-1}{\left(a_{r}^{3}-a_{r}^{1}\right)}^{T}\\ \cdots \\ \lambda_{1}^{-1}{\left(a_{r}^{q}-a_{r}^{1}\right)}^{T}\\ \lambda_{2}^{-1}{\left(a_{r}^{2}-a_{r}^{1}\right)}^{T}\\ \lambda_{2}^{-1}{\left(a_{r}^{3}-a_{r}^{1}\right)}^{T}\\ \cdots \\ \lambda_{2}^{-1}{\left(a_{r}^{q}-a_{r}^{1}\right)}^{T} \end{array}\right]$$

where $H_{r}$ represents the combination of the different satellites and different frequencies of LOS.

Then, the vector form of Equation (15) for different satellites and different frequencies can be written as:

(19) $$\Phi_{rb}=H_{r}b_{rb}$$

In consideration of the weighting for different carrier phase measurement, Equation (18) can be rewritten as:

(20) $$W\Phi_{rb}=WH_{r}b_{rb}$$

where W is the weighting matrix. In this work, the weighting matrix W is designed as two parts:

(21) $$W=W_{DDGF}W_{PLLt}$$

where $W_{DDGF}$ represents the DDGF detection weighting matrix and $W_{PLLt}$ is the weighting matrix based on PLL thermal noise. The DDGF detection weighting matrix is designed as:

(22) $$W_{DDGF}=\left[\begin{array}{cccccccc} p^{21} & & & & & & & \\ & p^{31} & & & & & & \\ & & \cdots & & & & & \\ & & & p^{q1} & & & & \\ & & & & p^{21} & & & \\ & & & & & p^{31} & & \\ & & & & & & \cdots & \\ & & & & & & & p^{q1} \end{array}\right]$$

where the element p is calculated by Equation (14). The DDGF detection weighting matrix is the identity matrix and will not influence the baseline vector result in case there is no large multipath or carrier phase noise. The corresponding carrier phase measurement will be eliminated in case its DDGF measurement is over the theoretical threshold.

As the PLL thermal noise is the main error source of the carrier phase error, in the case here, there is no large carrier phase noise or multipath [19]. In that case, we use the theoretical PLL thermal noise to determine the weighting. Assuming that the carrier phase noise between different receivers, satellites and frequencies is independent, the covariance matrix of the SD carrier phase measurement can be written as:

(23) $$D_{PLLt}^{SD}=\left[\begin{array}{cccccccc} {\left(\sigma_{PLLt,rb,1}^{1}\right)}^{2} & & & & & & & \\ & {\left(\sigma_{PLLt,rb,1}^{2}\right)}^{2} & & & & & & \\ & & \cdots & & & & & \\ & & & {\left(\sigma_{PLLt,rb,1}^{q}\right)}^{2} & & & & \\ & & & & {\left(\sigma_{PLLt,rb,2}^{1}\right)}^{2} & & & \\ & & & & & {\left(\sigma_{PLLt,rb,2}^{2}\right)}^{2} & & \\ & & & & & & \cdots & \\ & & & & & & & {\left(\sigma_{PLLt,rb,2}^{q}\right)}^{2} \end{array}\right]$$

where $D_{PLLt}^{SD}$ is the SD carrier phase measurement covariance matrix. Its element is calculated according to Equation (9).

The transportation matrix R from SD to DD can be expressed as:

(24) $$R=\left[\begin{array}{cccccccccc} -1 & 1 & & & & & & & & \\ -1 & & 1 & & & & & & & \\ \cdots & & & \cdots & & & & & & \\ -1 & & & & 1 & & & & & \\ & & & & & -1 & 1 & & & \\ & & & & & -1 & & 1 & & \\ & & & & & \cdots & & & \cdots & \\ & & & & & -1 & & & & 1 \end{array}\right]$$

Then, the DD carrier phase carrier phase measurement covariance matrix $D_{PLLt}^{DD}$can be calculated as follow:

(25) $$D_{PLLt}^{DD}=RD_{PLLt}^{SD}R^{T}$$

According to the error theory [4], the optimal weighting matrix can be determined as:

(26) $$W_{PLLt}={\left(D_{PLLt}^{DD}\right)}^{-1}$$

Finally, the weighted baseline vector can be calculated as Equation (27):

(27) $$\begin{array}{cc} b_{rb} & ={\left({\left(WH_{r}\right)}^{T}WH_{r}\right)}^{-1}{\left(WH_{r}\right)}^{T}W\Phi_{rb}\\ & ={\left(H_{r}^{T}W^{T}WH_{r}\right)}^{-1}H_{r}^{T}W^{T}W\Phi_{rb} \end{array}$$

After the baseline vector is known, the attitude can be calculated through a well-known and widely-used formula [30,38].

## 3. Theoretical Performance Analysis

In Section 2, the DDGF detection is proposed to detect large multipath and carrier phase noise for dual frequency carrier phase measurement. However, the DDGF detection is actually the carrier phase error difference checking between two frequencies. When the error in two frequencies is exactly the same, the proposed algorithm will fail. Therefore, the DDGF detection may not be suitable for all types of error. In this section, we will analyze the proposed algorithm theoretically. As the dual frequency characteristics are similar for different GNSS constellation, in this work, we choose B1 and B3 of the BeiDou navigation satellite System (BDS) and analyze its performance in detail. Meanwhile, the statistical results are listed for B1, B2 and B3 of BDS, L1, L2 and L5 of the Global Positioning System (GPS) and E1, E5 and E6 of Galileo. Table 1 shows the center frequencies of different GNSS.

**Figure 3:**
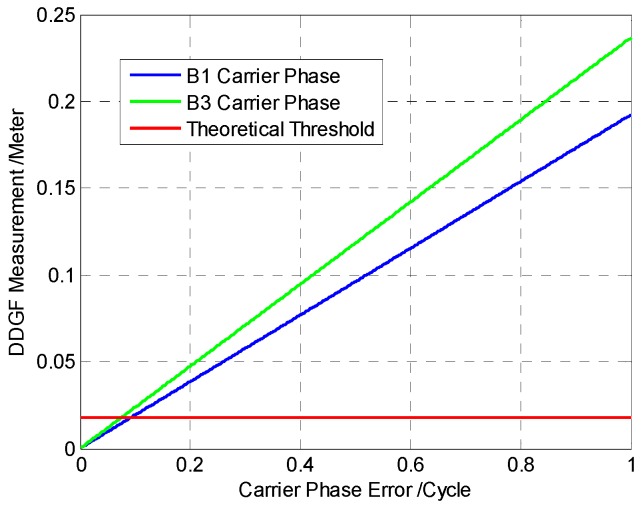
The relationship between the DDGF measurement and the carrier phase error in one frequency.

The DDGF measurement is linear with either of the dual frequency DD carrier phase according to the first line of Equation (7). Assuming only one of the two frequencies has error, Equation (7) can be treated as the linear equation with one variable. In that case, the DDGF measurement increases as the carrier phase error grows. Figure 3 shows the relationship between the DDGF measurement and the carrier phase error in one frequency. Meanwhile, the theoretical threshold is shown in Figure 3. The parameters for estimating the theoretical threshold are listed in Table 2.

As shown in Figure 3, the green line and blue line represent how DDGF measurement changes with the B1 and B3 carrier phase error. The DDGF measurement is far larger than the theoretical threshold when large error occurs. Therefore, the performance of DDGF detection is good in case only one frequency has large carrier phase error.

However, both frequencies may have large carrier phase error. Sometimes, the error in different frequencies may be the same. In that case, the difference of the dual frequency errors is close to zero. It makes the DDGF measurement below the theoretical threshold, but there may be large errors in both frequencies. Consequently, undetected error remains.

**Figure 4:**
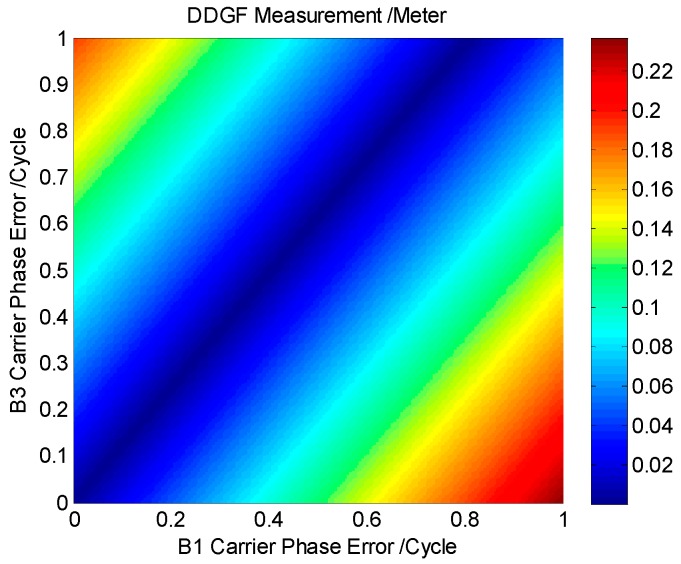
The relationship between the DDGF measurement and the carrier phase error in both frequencies.

Figure 4 shows the relationship between the DDGF measurement and the carrier phase error in both frequencies. The horizontal axis is the B1 carrier phase error, and the vertical axis is the B3 carrier phase error. The carrier phase measurement originally contains integer ambiguity. The integer ambiguity cannot be initialized in case the carrier phase error is larger than one cycle. Therefore, the B1 carrier phase error and B3 carrier phase error are between zero and one. Different colors represent the magnitude of the DDGF measurement. The magnitude is small in the color of blue and large in the color of red. It is clear that there is an area in dark blue where the B1 carrier phase error and B3 carrier phase error are both large, but the DDGF measurement is small. In this area, there will be undetected error after the DDGF detection.

**Figure 5:**
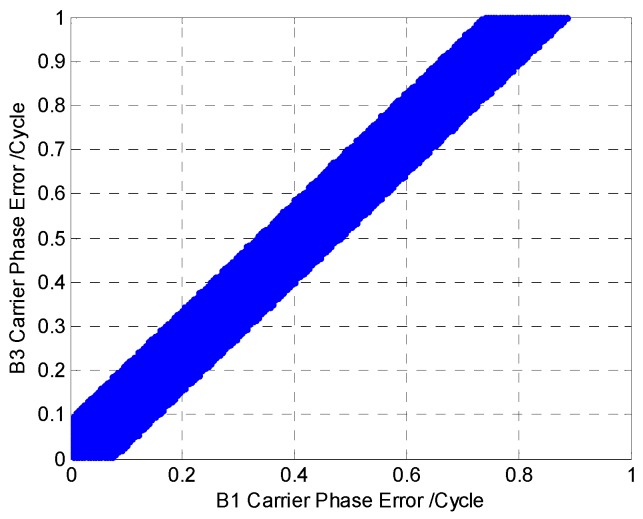
The DDGF detection failure area.

We change the color in Figure 4 into blue and white according to the theoretical threshold. Figure 5 shows the result. In the blue area, no matter how large the B1 and B3 carrier phase error is, the DDGF measurement cannot pass the theoretical threshold. In this work, we name it the DDGF detection failure area; on the contrary, the white area is the DDGF detection success area. The boundary of the DDGF failure area is related to the theoretical threshold. A lower theoretical threshold can make the blue area narrower; in other words, it can reduce the DDGF detection failure ratio. As the theoretical threshold is estimated by the receiver parameter and signal quality according to Equation (11), choosing a professional receiver and using the receiver in an open sky environment are good for DDGF detection and increase the DDGF detection success ratio.

We calculate the ratio of the DDGF detection success area out of the possible dual frequency error pairs. The result of B1-B3 is 85.30%. However, this result is based on the error being evenly distributed. Usually, the carrier phase error is not evenly distributed, as it can hardly reach one cycle. According to the previous work, the GNSS measurement error can be approximately treated as a zero mean with a Gaussian distribution [4,10]. Hence, we recalculate the success ratio for a zero mean Gaussian distributed error. The success ratio of B1-B3 is down to 73.96%. The reason that the success ratio is about 10% lower is meanly because the areas around (1,0) and (0,1) in the lower right and upper left corner of Figure 5 are very suitable for DDGF detection, as the dual frequency error difference is so large. However, the contribution of these areas to the result becomes very small when considering the error distribution. Then, we compute the success ratio for all of the GNSS constellation, and Table 3 shows the results. All of the GNSS constellation dual frequency results are about three quarters. This means that the DDGF detection can work with all of the GNSS constellation, and the success ratio is similar.

## 4. Experiments Analysis and Discussion

In order to demonstrate the proposed algorithm and evaluate its performance, a series of experiments were carried out in Beijing, China, including the open sky test, manmade multipath test and urban vehicle test.

### 4.1. Open Sky Test

**Figure 6:**
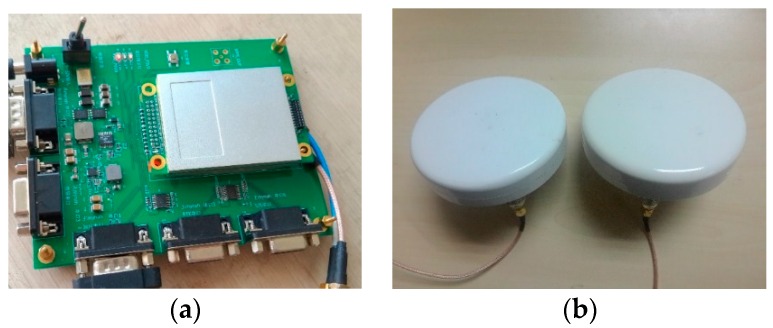
Experiment devices: (**a**) receiver; (**b**) antennas.

In this work, we choose B1 and B3 of BDS for the open sky test because the satellite type is various in BDS, including the Medium Earth Orbit (MEO), GEosynchronous Orbit (GEO) and Inclined GeoSynchronous Orbits (IGSO) satellites. The characteristic of all types of GNSS satellites can be analyzed. Two YWELL DY-GABR042P00D antennas and a RACOBIT OEM-B41 BDS dual frequency receiver were used. The receiver (a) and the antenna (b) are shown in Figure 6.

**Figure 7:**
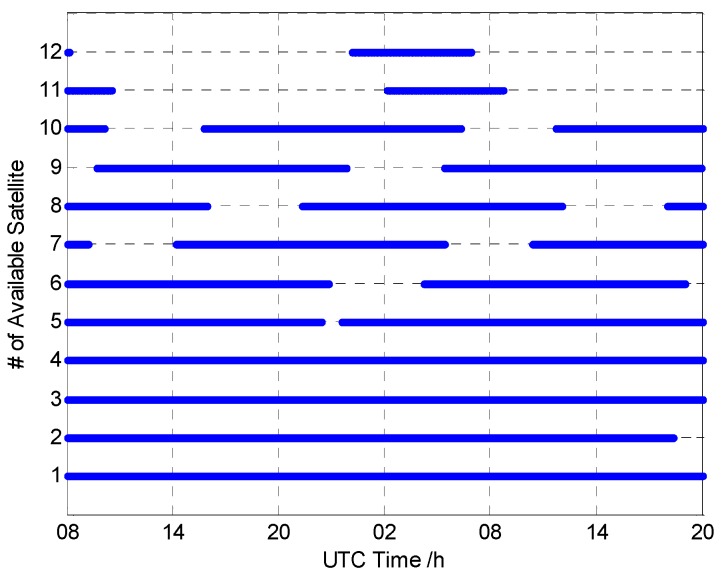
The available satellites of the collected data.

The data were collected at 0.1 Hz, with a five-degree cut-off elevation angle, from UTC time 08:02 to the next day 19:58, for a total of 12,930 epochs logged. Figure 7 shows the available satellites of the whole data. The blue line means the corresponding satellite is available. The satellites from #1 to #5 of BDS are GEO satellites. The GEO satellites are almost all available, as the GEO satellite is nearly static. The satellites from #6 to #10 of BDS are IGSO satellites. The orbit of the IGSO satellite is close to “8” at a certain longitude; hence, the IGSO satellites are available at the majority of time. The satellites #11 and #12 are MEO satellites. The orbit of the MEO satellite is global; hence, the available time is relatively short.

**Figure 8:**
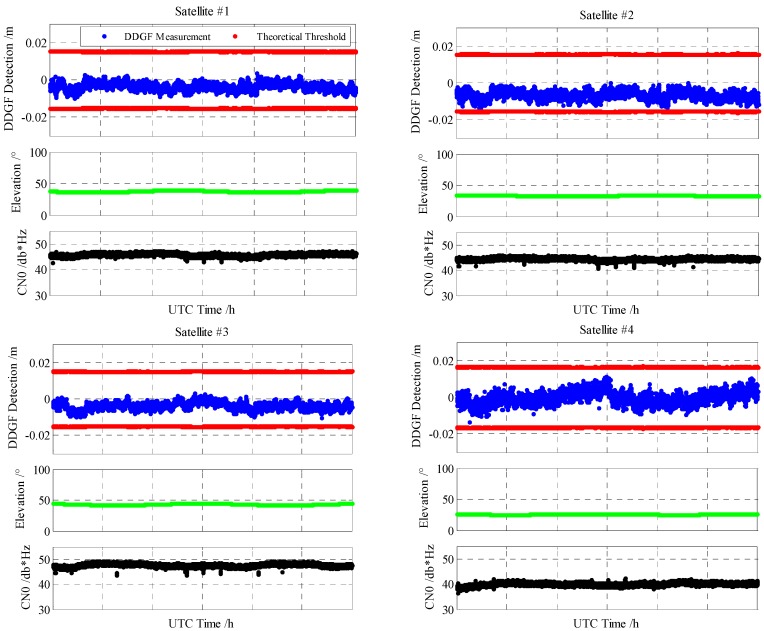

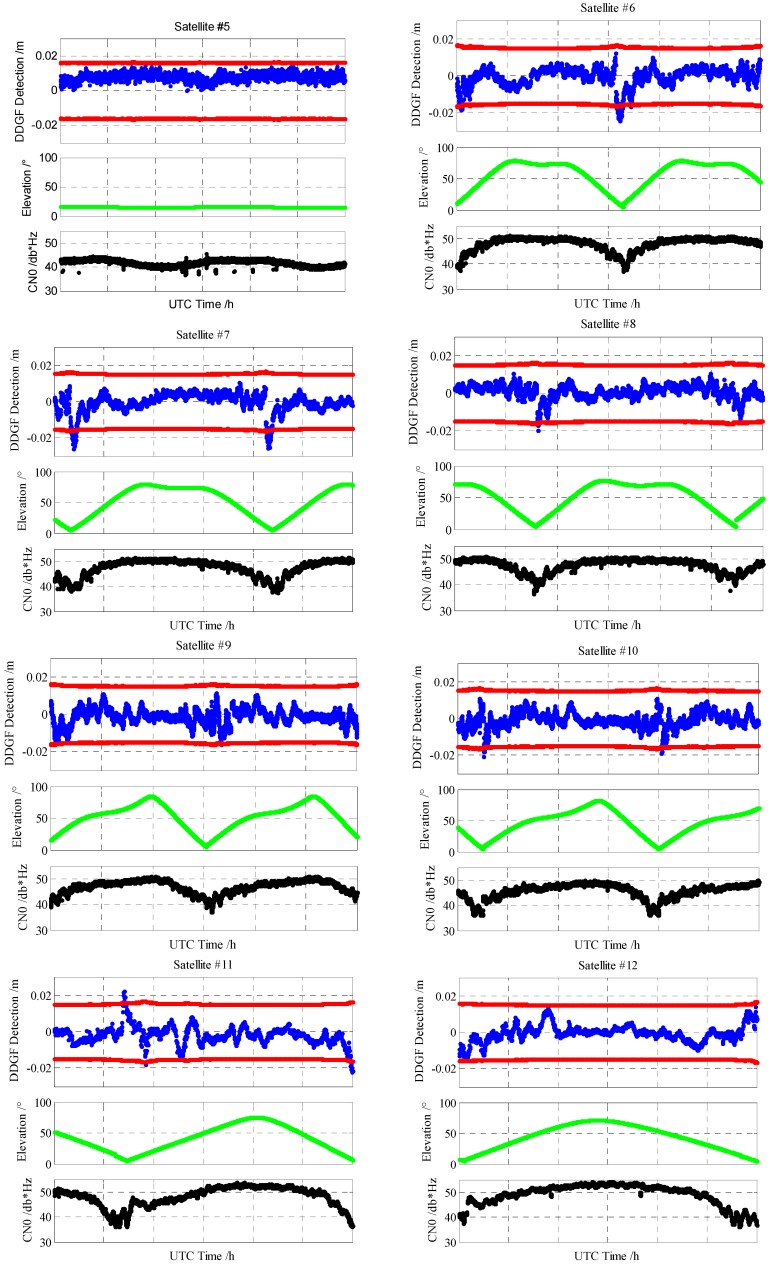
The DDGF detecting results together with the elevation angle and CN0.

Figure 8 is the proposed DDGF detecting results together with the elevation angle and the carrier to noise power expressed as a ratio (CN0) of all of the satellites. As the available satellites are discontinuous and there are no data for the unavailable period, the unavailable period is not shown in Figure 8. Then, the horizontal axis is not the same for different satellites. Hence, the horizontal axis is not labeled. The corresponding UTC time can be found in Figure 7.

From Figure 8, the elevation angle and CN0 of the GEO satellites from #1 to #5 are relatively stable as the GEO satellite is nearly static. As shown in the DDGF detection part in Figure 8, the blue line is the DDGF measurement, and the red lines represent the theoretical threshold. The DDGF measurement of GEO satellites is stable, but obviously not zero mean. The satellite of a low elevation angle has a relatively large bias from zero, and the noise of the low CN0 satellite is relatively large. It meets the supposed results theoretically that the multipath in the low elevation angle satellite is large and that the carrier phase noise in the low CN0 satellite is large. The result indicates that although the environment of the open sky test is good, the multipath still exists. Obviously, the GEO multipath cannot be canceled from averaging the long-term data. Fortunately, the DDGF measurement of the open sky test is within the theoretical threshold. This means the multipath is within its regular value and will not influence the accuracy very much.

As for the IGSO and MEO satellites, the DDGF detecting results are similarly. Different from the GEO satellite, the DDGF measurement is nearly zero mean and varies greatly. It may be larger than the theoretical threshold in the case that the elevation angle is very low.

**Figure 9:**
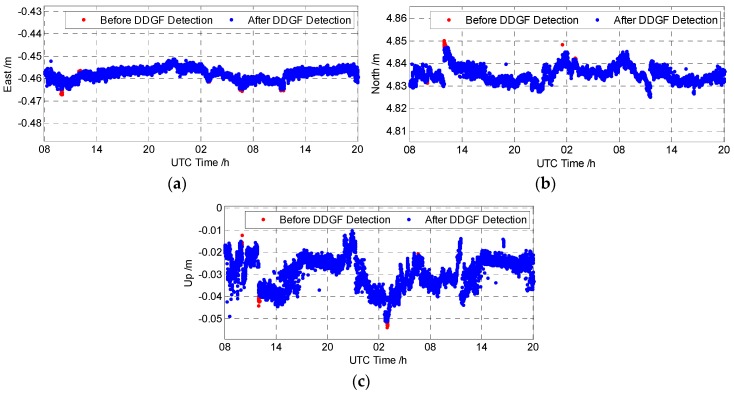
The baseline vector of the open sky test (**a**) East; (**b**) North; (**c**) Up.

Figure 9 shows the carrier phase relative positioning result, namely the baseline vector in the east, north and up directions. The red dots in the back layer represent the baseline result before DDGF detection, and the blue dots in the front layer are the baseline result after DDGF detection. The baseline vector before and after DDGF detection are coincident most of the time. As some carrier phase measurements with large error are eliminated after DDGF detection, the corresponding large baseline errors become smaller compared with the baseline before DDGF detection in red. This proves that the proposed algorithm is able to mitigate the occasional large baseline error in the open sky environment.

### 4.2. Manmade Multipath Test

**Figure 10:**
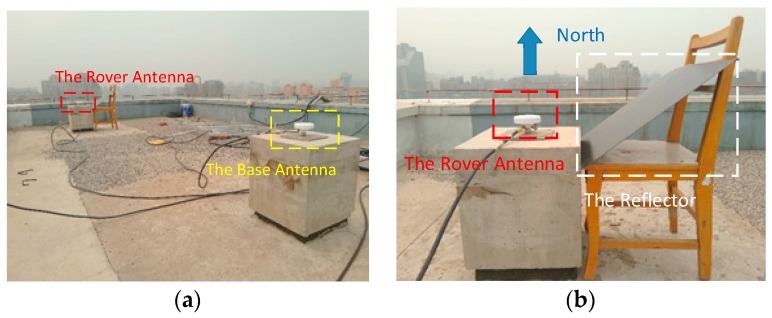
The multipath environment: (**a**) the location of the two antennas; (**b**) the illustration of the reflector.

The manmade multipath test is analyzed to evaluate the performance of DDGF detection in severe multipath environments. The same baseline, experiment devices and frequencies are used in the manmade multipath test compared to the open sky test. Hence, the mean value of the previous 36-h open sky test result can be used as the reference. A reflector was set to the east side of the rover antenna. The multipath environment is shown in Figure 10.

**Figure 11:**
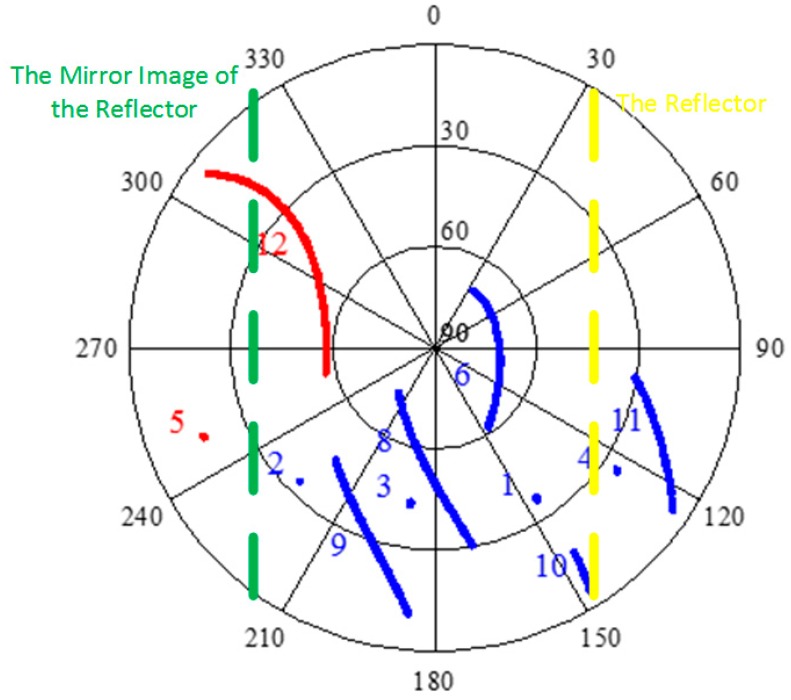
The reflector and the sky plot of the satellites.

The data were collected at 1 Hz, with a five degree cut-off elevation angle, from UTC time 23:01 to the next day 05:00 for a total of 21,580 epochs logged. Figure 11 is the sky plot of the satellites and the geometrical relationship between the satellites and the reflector. The azimuth and elevation angles for all of the available satellites are computed and plotted in polar coordinates. From Figure 11, the satellite #7 is not available during the test. Theoretically, the signal of the satellites opposite the reflector will suffer from the multipath more severely. These kinds of satellites are marked in red in Figure 11.

**Figure 12:**
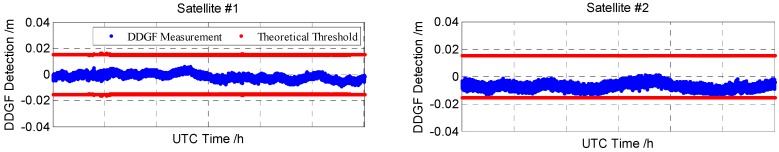

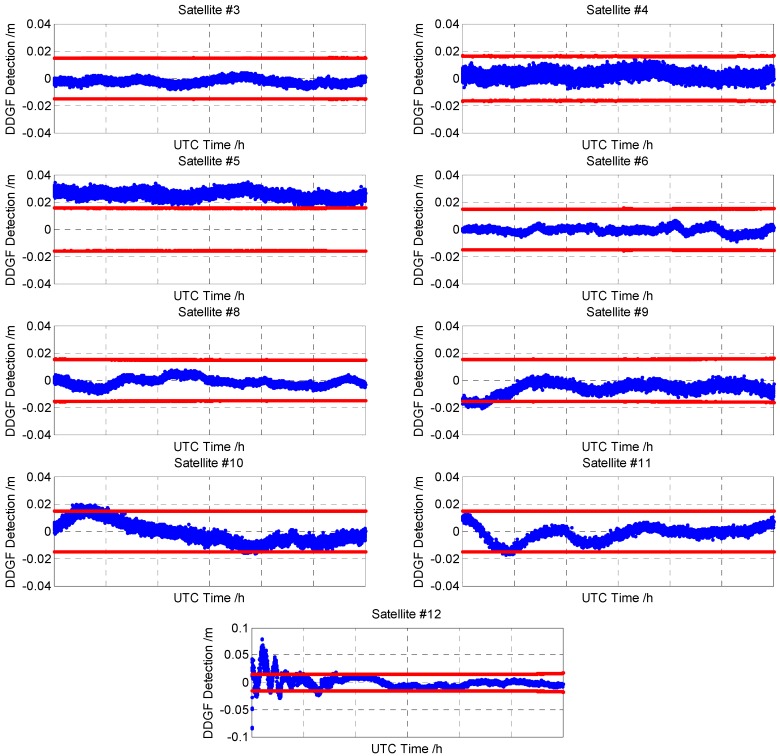
The DDGF detecting results of the manmade multipath test.

The results of the DDGF detection are shown in Figure 12. Compared with Figure 8, the characteristic is similar. The DDGF measurement of GEO satellites is stable, but not zero mean, and the DDGF measurement of IGSO and MEO varies greatly. Satellite #5 is influenced severely, and its DDGF measurement is above the theoretical threshold during the whole test. Satellite #12 arises from the northwest corner with a low elevation angle. Its DDGF measurement is much larger than the theoretical threshold when its elevation is low. As the elevation angle increases, the DDGF measurement is back to normal. 

**Figure 13:**
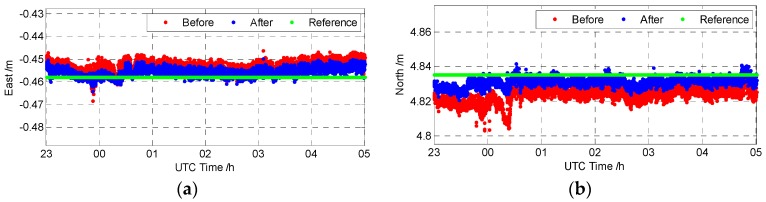

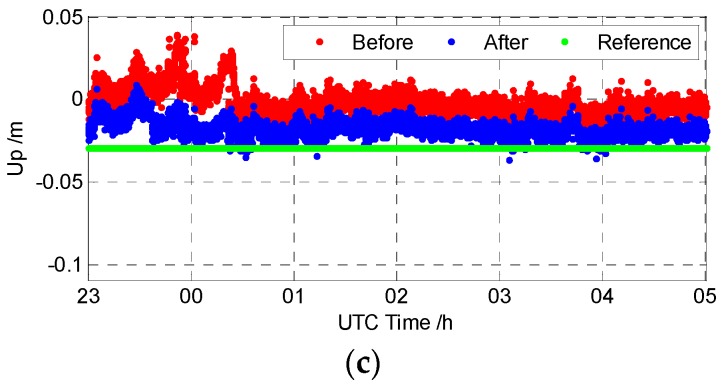
The baseline vector of the manmade multipath test (**a**) East; (**b**) North; (**c**) Up.

Figure 13 shows the baseline results before and after DDGF detection together with the reference result. In Figure 13, “Before” and “After” respectively represent the baseline results before and after DDGF detection. The mean value of the previous 36-h open sky test is treated as the reference result, namely the “Reference” in Figure 13. It is clear that the baseline result after the DDGF detection is closer to the reference value. Meanwhile, the large error at about zero hours UTC time is mitigated in the baseline result after DDGF detection. Table 4 is the mean value of the baseline before and after DDGF detection and the reference baseline.

**Figure 14:**
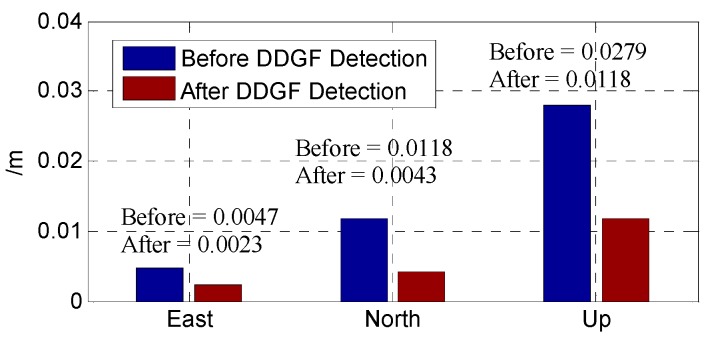
The baseline error compared with the reference.

Figure 14 is the baseline error before and after DDGF detection compared with the reference. The “Before” is the difference of the mean value between the baseline before DDGF detection and the reference baseline, and so as the “After”. The baseline error in up is influenced the most by the manmade multipath. The error in north is worse than that in east. The error in these three directions matches the DOP (Dilution of Precision) of BDS in China, as the GEO satellites are located in a line in the east-west direction [31]. As shown in Figure 14, after the DDGF detection, the baseline error after DDGF detection is about a half of the baseline error before the DDGF detection compared with the long-term open sky reference baseline.

### 4.3. Urban Vehicle Test

**Figure 15:**
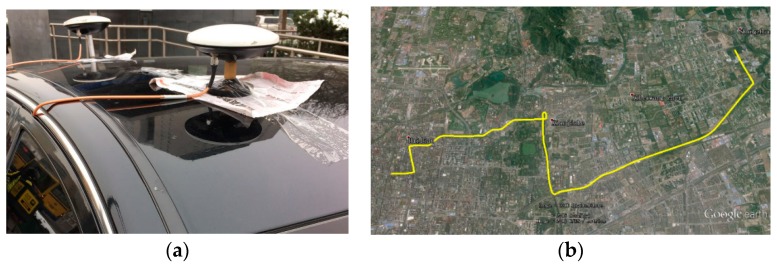
The urban vehicle test condition: (**a**) the location of two antennas; (**b**) the route of the urban vehicle test.

The urban vehicle test was carried out to demonstrate the dynamic performance of the proposed algorithm. This time, GPS L1 and L2 are chosen in consideration that the satellite distribution of GPS is more suitable for blocking from all directions. Two HARXON HX-CS3601A survey antennas and NovAtel-OEM628 dual frequency receivers were used. The location of two antennas is shown in Figure 15a.

As the true baseline vector is unable to be obtained in a dynamic situation, we use the baseline length to evaluate the performance because the baseline length is unchanged no matter how the vehicle moves. Before driving, the baseline length is measured from the 10-hour static open sky average value.

The driving was in Haidian District, Beijing, China. The driving route is shown in Figure 15b.

**Figure 16:**
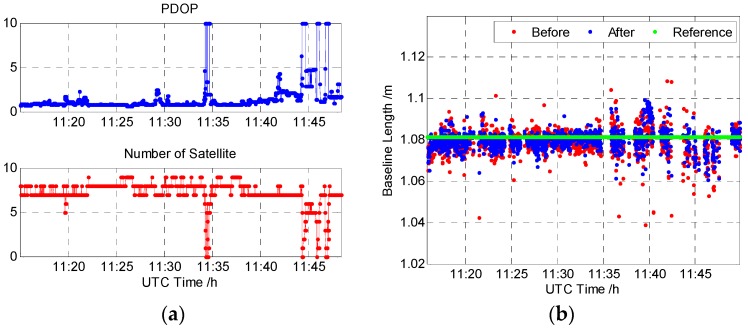
The urban vehicle test result: (**a**) the Position Dilution of Precision (PDOP) and the total satellite number; (**b**) the baseline length of the urban vehicle test.

Data were collected at 1 Hz, with a five degree cut-off elevation angle, between 11:15 and 11:50, UTC time, for a total of 2592 epochs logged. Figure 16a shows the number of satellites tracked and the Position Dilution of Precision (PDOP). The number of tracked satellites is about seven to eight most of the time. At around 11:35 and 11:45, the number of satellite drops down severely, even to zero. PDOP is around two most of the time. Similarly, at around 11:35 and 11:45, PDOP severely increases up to ten.

Figure 16b is the baseline length of the urban vehicle test. The baseline length error is relatively stable and small when the PDOP is low before 11:35 UTC time. When the PDOP gets higher, the baseline error becomes larger. The baseline length results before and after DDGF detection are around the standard, and they are at the same magnitude level at most of the time. Some large baseline length errors above 0.03 m are mitigated by the DDGF detection in the proposed results. The baseline length calculated by the proposed algorithm is more stable. Table 5 shows the statistic results. The column “Baseline Length” is the mean value of the baseline length. The mean value of the baseline after DDGF detection is closer to the reference. The bias decreases from 0.008 m to 0.002 m. The standard deviation of the baseline length after DDGF detection is smaller than that before DDGF detection by about 8%. The results indicate that the proposed algorithm improves both the mean value and the standard deviation performance in the urban vehicle test.

## 5. Conclusions

In this work, we propose a dual frequency carrier phase error difference checking algorithm for the GNSS compass. The algorithm aims at eliminating large carrier phase noise and multipath error in DD carrier phase measurement. We utilize the characteristic that the different frequency carrier phase measurements contain the same geometrical distance and form the DDGF measurement. The DDGF detection is designed according to the theoretical estimation of the carrier phase error. The theoretical performance of the proposed DDGF detection is analyzed. The result shows that the detecting success ratio of all of the GNSS constellation dual frequency pairs is about three quarters in the condition that the error is zero mean Gaussian distributed.

We analyze the characteristic of the DDGF measurement in different types of satellites from the open sky test. The DDGF measurement of GEO satellites is stable, but not zero mean. As for IGSO and MEO, it is nearly zero mean, but varies greatly and may be larger than the theoretical threshold in the case that the satellite elevation angle is very low. Some large errors of the baseline vector can be mitigated after the DDGF detection.

The manmade multipath test is analyzed to evaluate the performance of DDGF detection in a severe multipath environment. As the location of the reflector is known, the satellites that may be suffering from the multipath severely are known. The DDGF detection result matches the anticipation. The DDGF measurement of the influenced GEO satellite is above the theoretical threshold during the whole test. The DDGF measurement of the influenced MEO satellite is much larger than the theoretical threshold when its elevation is low. As the elevation angle increases, the DDGF measurement is back to normal. After the DDGF detection, the baseline vector error is about a half of the result before DDGF detection compared with the long-term open sky reference baseline vector.

The urban vehicle test was carried out to evaluate the dynamic performance of the proposed algorithm. We analyze the baseline length because the baseline length is unchanged no matter how the vehicle moves. The 10-h static open sky average value is treated as the reference. The results indicate that the proposed algorithm improves both the mean value and the standard deviation performance in the urban vehicle test.

Based on these results, the proposed DDGF detection has the ability to detect large error in dual frequency carrier phase measurement without the help of prior environment information. After the DDGF detection, the accuracy of the baseline vector is improved in the GNSS compass.

## Figures and Tables

**Table 1:** The center frequencies of different GNSS. BDS, BeiDou navigation satellite System.

GNSS Name	Frequency Band	Center Frequency/MHz
BDS	B1	1561.098
	B2	1207.14
	B3	1268.52
GPS	L1	1575.42
	L2	1227.6
	L5	1176.45
Galileo	E1	1575.42
	E5a	1176.45
	E5b	1207.14
	E6	1278.75

**Table 2:** The parameters for estimating the theoretical threshold.

**Parameter**	$B_{n}$/Hz	T/ms	$C/N_{0}$/dB*Hz	$\sigma_{A}\left(\tau \right)$	$\sigma_{v}$/°
**Typical Value**	10	1	40	$1\times 10^{-10}$	2

**Table 3:** The ratio for which the DDGF detection is able to work.

GNSS Name	Frequency Pair	Success Ratio/%
BDS	B1-B3	73.96
	B1-B2	74.29
	B2-B3	74.96
GPS	L1-L2	74.15
	L2-L5	75.19
	L1-L5	74.41
Galileo	E1-E5a	74.41
	E1-E5b	74.27
	E1-E6	73.90
	E5a-E5b	75.20
	E5a-E6	75.05
	E5b-E6	74.94

**Table 4:** The mean value of different baseline results.

Result Type	East/m	North/m	Up/m
Reference Baseline	−0.4578	4.8354	−0.0292
Baseline Before DDGF Detection	−0.4531	4.8236	−0.0013
Baseline After DDGF Detection	−0.4555	4.8311	−0.0174

**Table 5:** The statistic results of different baseline lengths in the urban vehicle test.

Result Type	Baseline Length/m	Bias to the Standard Length/m	Standard Deviation/m
Reference Baseline	1.0797	-	-
Baseline Before DDGF Detection	1.0789	0.008	0.0061
Baseline After DDGF Detection	1.0795	0.002	0.0056
